# Unusual cause of exercise-induced ventricular fibrillation in a well-trained adult endurance athlete: a case report

**DOI:** 10.1186/1752-1947-2-120

**Published:** 2008-04-23

**Authors:** Stefan Vogt, Daniel Koenig, Stephan Prettin, Torben Pottgiesser, Juergen Allgeier, Hans-Hermann Dickhuth, Anja Hirschmueller

**Affiliations:** 1 University of Freiburg, Department of Preventive and Rehabilitative Sports Medicine, Germany; 2 Interventional Cardiology, Herz-Zentrum, Bad Krozingen, Germany

## Abstract

**Introduction:**

The diseases responsible for sudden deaths in athletes differ considerably with regard to age. In young athletes, congenital malformations of the heart and/or vascular system cause the majority of deaths and can only be detected noninvasively by complex diagnostics. In contrast, in older athletes who die suddenly, atherosclerotic disease of the coronary arteries is mostly found. Reports of congenital coronary anomalies as a cause of sudden death in older athletes are rare.

**Case presentation:**

A 48-year-old man who was a well-trained, long-distance runner collapsed at the finish of a half marathon because of a myocardial infarction with ventricular fibrillation. Coronary angiography showed an anomalous origin of the right coronary artery from the left sinus of Valsalva with minimal wall alterations. Multislice computed tomography of the coronary arteries confirmed these findings. Cardiomagnetic resonance imaging demonstrated a mild hypokinesia of the basal right- and left-ventricular posterior wall. An electrophysiological study showed an inducible temporary polymorphic ventricular tachycardia and an inducible ventricular fibrillation. The athlete was subsequently treated by acetylsalicylic acid 100 mg (0-1-0), bisoprolol 2.5 mg (1-0-0) and atorvastatin 10 mg (0-0-1) and was instructed to keep his training intensity under the 'individual anaerobic threshold'. Intense and long-lasting exercise under extreme environmental conditions, particularly heat, should also be avoided.

**Conclusion:**

This case report presents a coronary anomaly as the most likely reason for an exercise-induced myocardial infarction with ventricular fibrillation in a well-trained 48-year-old endurance athlete. Therefore, coronary anomalies have also to be considered as a possible cause of cardiac problems in older athletes.

## Introduction

Sudden death has been defined as "an abrupt unexpected death of cardiovascular cause, in which the loss of consciousness occurs within 1 to 12 hours of onset of symptoms" [1]. Although sudden deaths in athletes are dramatic and tragic occurrences, the total incidence of sudden death during sport is rather low. The annual incidence of sudden deaths in athletes under 35 years is 2.62 per 100,000 for male and 1.07 for female athletes [2], whereas the risk of sudden death in athletes over 60 years old can be 100-fold higher compared with young athletes [3]. The precise diseases responsible for sudden death differ considerably with regard to age. In young athletes, congenital malformations of the heart and/or vascular system cause the majority of deaths and can only be detected noninvasively by complex diagnostics [4,5]. In contrast, the underlying cause in older athletes who die suddenly is usually atherosclerosis of the coronary arteries [3]. Reports of congenital coronary anomalies as a cause of sudden death in older athletes are rare.

This case report presents a coronary anomaly as the most likely reason for an episode of exercise-induced ventricular fibrillation in a well-trained 48-year-old endurance athlete.

## Case presentation

A 48-year-old, well-trained, long-distance runner collapsed at the finish of a half marathon. On the day of the incident, the air temperature was relatively high (25°C). Against his usual practice, the athlete tried to accelerate on the last hundred meters towards the finish line. Immediately after the collapse, cardiopulmonary resuscitation with defibrillation of ventricular fibrillation was successfully carried out.

There were no indications of cardiovascular and other serious diseases in the athlete's medical history. The athlete had never noticed any cardiac symptoms, in particular no anginal discomfort, dysrhythmia or episodes of cardiac syncope. Since youth, regular endurance training had been performed without any problems. Before the incident, his training load was 30 to 40 kilometres of running each week. The athlete's family history was also negative for cardiovascular diseases. He was taking no regular medication.

The patient was a 48-year-old man, stature 178 cm, body mass 83.6 kg, blood pressure (BP) 120/70 mmHg, heart rate 48/minute. Percussion and auscultation of the heart and lung showed no pathological findings. His troponin T level was slightly elevated in the emergency room (0.069 ng/ml), and significantly elevated 1 day after the myocardial infarction (0.392 ng/ml). It returned to normal levels within 5 days. An electrocardiogram (ECG) was performed and showed a normal axis and sinus rhythm, heart rate 65/minute, and no pathological findings. Echocardiography showed a normal configuration of the four chambers, with good left and right ventricular function.

Laboratory findings showed elevated total cholesterol (217 mg/dl) and low-density lipoprotein (LDL)-cholesterol (161 mg/dl) levels, and reduced high-density lipoprotein (HDL)-cholesterol (47 mg/dl) levels. All other laboratory findings, in particular the electrolyte levels, were within normal ranges.

**Figure 1:**
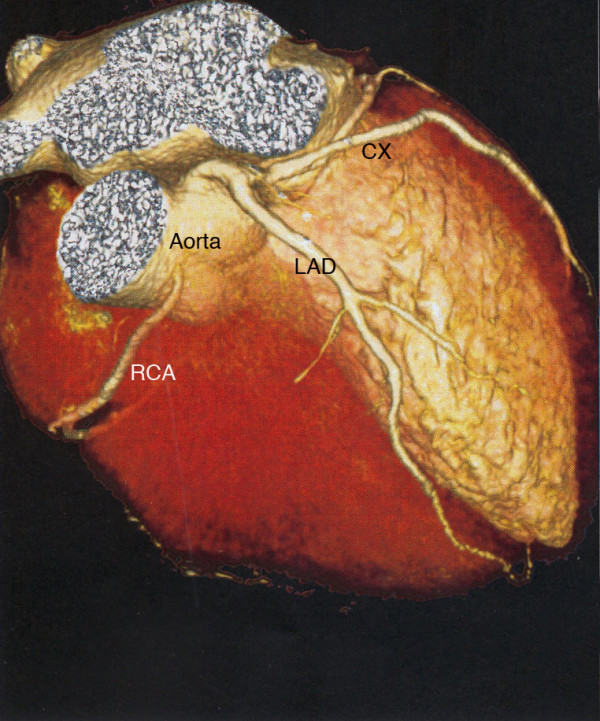
**Multislice computed tomography of the heart demonstrates the coronary artery anomaly with a left-side origin of the right coronary artery**. CX, circumflex coronary; LAD, left anterior descending artery; RCA, right coronary artery. The right ventricle has been digitally removed.

**Figure 2:**
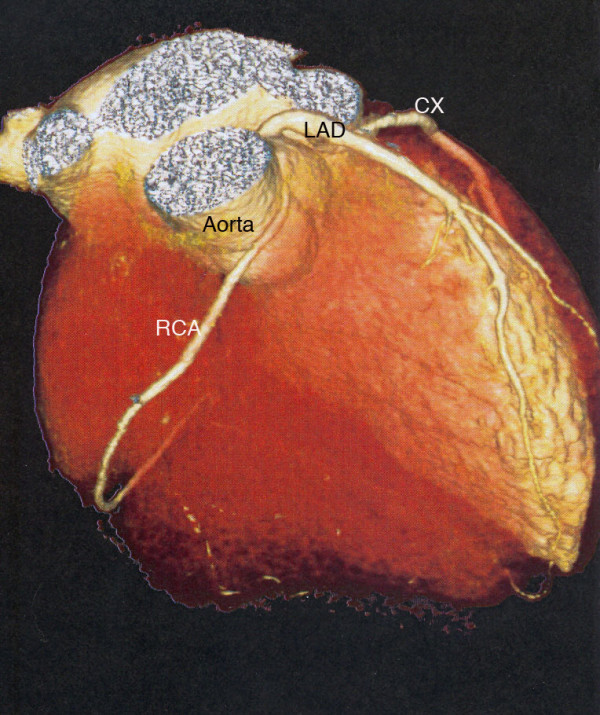
**Multislice computed tomography of the heart demonstrates the coronary artery anomaly with a left-side origin of the right coronary artery**. CX, circumflex coronary; LAD, left anterior descending artery; RCA, right coronary artery. The right ventricle has been digitally removed.

**Figure 3:**
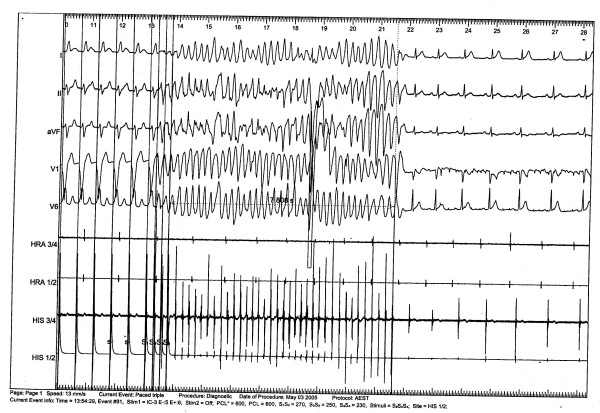
An electrocardiogram performed during the electrophysiology study showed an inducible temporary ventricular fibrillation (duration: 8 seconds).

A coronary angiography was conducted because of the unknown etiology of the ventricular fibrillation. A coronary artery anomaly with a left-side origin of the right coronary artery (RCA) with minimal wall alterations was revealed. Multislice computed tomography of the coronary arteries confirmed these findings (Figures 1 and 2). Cardio-magnetic resonance imaging demonstrated a non-transmural late-enhancement of gadolinium in the basal ventricular posterior wall, on both right and left sides, with corresponding hypokinesia of the right ventricular wall and an ejection factor of 48%. Two days after the incident, an electrophysiology study showed inducible temporary polymorphic ventricular tachycardia and inducible ventricular fibrillation (Figure 3). The electrophysiology study was performed at the apex of the right ventricle (base-stimulation: 600 ms, extra-stimulations beginning with 250 ms). The myocardial scar could have been responsible for the induction of ventricular fibrillation. However, this finding was unspecific and did not completely clarify the etiology of the symptoms. No cardiac arrhythmia was detected in a 24-hour ECG.

Subsequently, the patient was treated with acetylsalicylic acid 100 mg (0-1-0), bisoprolol 2.5 mg (1-0-0), atorvastatin 10 mg (0-0-1) and potassium chloride (1-1-1).

Four months later, the athlete performed an incremental cycling and running test until complete exhaustion and demonstrated good endurance capacity. An ECG performed at rest and during exercise showed no pathological findings. Echocardiography showed normal heart configuration and good ventricular function.

Laboratory findings were normal, in particular, normal plasma glucose levels and normal triglyceride and cholesterol levels (HDL-cholesterol 54 mg/dl; LDL-cholesterol 115 mg/dl; triglycerides 168 mg/dl).

At a 1-year follow-up examination, his body mass had increased by 3 kg and his total cholesterol level was elevated (256 mg/dl; HDL-cholesterol 47 mg/dl; LDL-cholesterol 171 mg/dl; triglycerides 181 mg/dl). The cardiologic examinations, in particular the ECG during exhausting treadmill exercise, showed no pathological findings.

## Conclusion

The majority of sudden deaths in athletes occur during or immediately after exercise. However some deaths occur at rest or during sleep. Cardiovascular preparticipation screening is an essential procedure to diagnose any underlying cardiovascular abnormalities that may predispose an athlete to sudden death. There has been controversial discussion of the potential of preparticipation screening to prevent sudden deaths in athletes [6-10].

In this case report, all examinations routinely carried out in preparticipation screening were normal. Apart from minimal wall alterations, the coronary system showed no significant stenosis. Thus, the anomalous origin of the RCA with its unusual anterior and acute-angled course between the ascending aorta and the right ventricular outflow tract probably contributed to the myocardial infarction [11]. The increased myocardial oxygen demand during exercise and a potential mechanical obstruction of the RCA through pulsation of the aorta and right ventricular outflow tract might have negatively influenced the myocardial perfusion. It can be speculated that the intramural cardiac ischemia in the posterior wall triggered the ventricular fibrillation.

Until now, this coronary artery anomaly has not been considered as a pathologic anomaly [5,12] because the RCA mostly does not run between the aorta and pulmonary artery. However, other authors [13-15] have assumed that this anomaly can cause sudden death, especially in young athletes. The risk for older athletes is as yet unknown.

A bypass operation did not seem appropriate for this patient because of an expected steal-mechanism owing to a relatively wide arteria mammaria interna and a comparatively thin RCA. To lessen the risk of a relapse, the implantation of an automatic implantable cardioverter defibrillator (AICD) was indicated. The athlete disapproved of the AICD implantation. He was recommended to maintain his medication and instructed to keep his moderate training intensity under the so-called 'individual anaerobic threshold' [16]. As the incident happened under extreme environmental conditions, the patient was advised that intense and long-lasting exercise in such conditions, particularly exercising in heat, should be avoided. The implantation of an AICD was postponed for a trial period.

## Abbreviations

AICD: automatic implantable cardioverter defibrillator; CX: circumflex coronary; ECG: electrocardiogram; HDL: high-density lipoprotein; LAD: left anterior descending artery; LDL: low-density lipoprotein; RCA: right coronary artery.

## Competing interests

The authors declare that they have no competing interests.

## Authors' contributions

SV, DK, SP, TP and AH were involved in the conception, design, drafting and revising of the manuscript. JA, DK and HHD were involved in the diagnosis and treatment of the patient and in revising the manuscript. All authors read and approved the final manuscript.

## Consent

Written informed consent was obtained from the patient for publication of this case report and any accompanying images. A copy of the written consent is available for review by the Editor-in-Chief of this journal.
